# Superior Vena Cava Syndrome and Otorrhagia During Cardiac Surgery

**DOI:** 10.7759/cureus.4602

**Published:** 2019-05-05

**Authors:** Azfar K Niazi, Alexandra S Reese, Paul Minko, Donal O'Donoghue, Sabry Ayad

**Affiliations:** 1 Outcomes Research, Cleveland Clinic, Cleveland, USA; 2 Miscellaneous, St. George's University, St. George's, GRD; 3 Anesthesiology, Cleveland Clinic Fairview Hospital, Cleveland, USA

**Keywords:** superior vena cava syndrome, otorrhagia, coronary artery bypass graft

## Abstract

Otorrhagia during cardiac surgery is rare. Otorrhagia combined with other signs of increased venous pressure in the upper body indicates the development of superior vena cava (SVC) syndrome. In this case, ear bleeding, facial engorgement, and conjunctival edema were noticed. The SVC cannula was displaced, leading to SVC syndrome. Repositioning of the cannula led to rapid recovery of the symptoms and an uneventful postoperative course. Providers should be vigilant about signs of SVC obstruction. Transparent coverings and surgical shelves should be used for constant examination of the head and neck to immediately detect changes.

## Introduction

Superior vena cava (SVC) syndrome is a result of obstruction of flow through the SVC, the major venous outlet for deoxygenated blood from the head, neck, and upper torso to the heart. The SVC is relatively pliable and can be easily compressed by a mass or an intravascular device. The consequent pressure increase in the SVC can produce edema of the head, neck, and arms. It is often accompanied by cyanosis, distension of the veins of the upper body, and dyspnea. Symptom severity is generally worse when obstruction develops rapidly [1].

Cannulae are used during cardiac surgery to divert blood from the superior and inferior vena cavae to the heart-lung machine for oxygenation and then back to the ascending aorta [2]. Small deviations in the cannula position can obstruct venous blood flow. If the cannula is displaced in the SVC it can result in SVC syndrome. A rare consequence of SVC syndrome during cardiac surgery is otorrhagia [3].

## Case presentation

A 59-year-old male presented with a history of stroke and diagnosed with streptococcus mitis-oralis endocarditis caused by a recent tooth infection. The patient had an infected mitral valve with vegetations on both leaflets and was treated with intravenous antibiotics but later developed rapid atrial flutter, tachycardia, and mitral valve regurgitation. The condition was severe enough for the patient to undergo a mitral valve replacement.

At the start of the procedure, an aortic and bicaval venous cannulation was carried out and tapes were passed around the cannulae. The aorta was cross-clamped with a soft padded clamp. Cold blood cardioplegia was given to arrest the heart. Within 10 minutes after cross-clamping, central venous pressure rose from 5 mmHg to 30 mmHg which coincided with the application of the tapes to the SVC syndrome. Cerebral oximetry values decreased significantly from pre-pump values of 67% in the left and 61% in the right to 44% in the left and 45% in the right shortly after the patient was placed on cardiopulmonary bypass (CPB) machine. The perfusionist was alerted to these changes. There were no issues with the functionality of the CPB machine. The patient was noted to have spontaneous bleeding from the left ear. The examination was difficult because of the metal surgical shelf covering the patient’s head. The face and head were swollen, and conjunctival edema made the examination of the pupils difficult. However, they were determined to be small and reactive to light. SVC syndrome was diagnosed and the SVC cannula was noted to be displaced. Within minutes of repositioning it, the facial swelling resolved and the central venous pressure decreased to 5 mmHg. Cerebral/somatic oximetry increased to 52% on the left side and 58% on the right side. The bispectral index remained at 35-45 throughout the CPB. The surgical procedure ended uneventfully.

On admission to the intensive care unit, no acute bleeding was seen in either of the ears but dried blood was seen on external auditory meatus of the left ear. The patient was awakened soon after he was admitted to the surgical intensive care unit. No neurological deficits were noted. There was no further otorrhagia after surgery. Ear exams performed immediately after the surgery and again a week after the surgery showed minimal dried blood in the left ear. The tympanic membrane was intact. No fluid was noticed in the middle ear cleft. No erythema or edema was noticed in either of the ears.

## Discussion

After noticing the bleeding ear and hemodynamic changes on the monitors, the surgical team was promptly notified and the etiology of the changes was found and corrected. This prompt response resulted in an excellent recovery with no further sequelae. The metal surgical shelf limited the ability of the anesthesia team to notice the obvious changes in the face due to the build-up of pressure in the upper body venous system. An uncommon symptom of SVC syndrome is a bleeding ear which apparently results from several vascular pathways that become engorged when the SVC is largely obstructed. Otorrhagia results when there is insufficient time for the vessels to adjust to an acute increase in back pressure in the venous system which in turn increases pressure in subcutaneous capillaries in the external ear.

Otorrhagia has not been reported during cardiac surgery, but Singh et al. reported a similar case of a patient who had otorrhagia at the end of mitral valve replacement surgery. The patient had prolonged elevated central venous pressure during the surgery [3].

Cardiac and thoracic surgery often involves extensive manipulation of intra-thoracic structures which increases susceptibility to SVC syndrome as well. A case of acute SVC syndrome during cardiac surgery was reported by Amundson et al. [4]. During the surgery, the left internal thoracic artery (LITA) retractor was placed. After the LITA retractor placement, central venous pressure (CVP) increased and the patient developed significant scleral edema. The patient’s head became engorged and plethoric. Systemic pressures were declining and mid-esophageal view on transesophageal echocardiography showed complete SVC syndrome compression leading to confirmation of acute SVC syndrome diagnosis.

During cardiac surgery, SVC syndrome can remain undiagnosed because of limited visibility of the patient’s face and body. In our hospital, a metal surgical shelf is placed over the patient’s head to prevent injury and for placing surgical instruments. It played an important role in our case because it impeded anesthesia providers’ access to the patient's head and face which contributed to a delay in making the diagnosis. If the patient’s face had been easily visible throughout the procedure, the facial swelling would likely have been observed earlier. Another case of an eight-month-old infant with mitral regurgitation and coarctation of aorta undergoing cardiac surgery, reported by Shibasaki et al., had increased CVP after weaning from CPB despite the positive inotropes administration. The reason for high CVP could not be found until the drapes were taken off revealing the difference in the skin color between upper and lower body [5].

SVC syndrome has been observed in the early postoperative period after cardiac surgery. Rossi and Manica published a case in which an infant who had a repair of sinus venous atrial septal defect was found to have differential cyanosis on the first postoperative day leading to the diagnosis of SVC syndrome possibly secondary to extrinsic compression due to the inflammatory reaction and local edema. It was successfully treated by percutaneous angioplasty with covered-stent implantation [6].

There are other etiologies which mimic/cause the clinical picture of SVC syndrome. Echocardiography is very useful in diagnosing the exact cause of the venous back pressure. A case of a four-month-old infant who had ventricular septal defect repair reported by Pierli et al., developed jugular venous distention with bluish discoloration and edema in her face, arm, and upper trunk after the surgery. The chest radiograph was non-diagnostic. The hemodynamic status deteriorated resulting in a decrease in the systemic pressure despite dopamine infusion and fluid administration along with a pulse of 200 beats per minute with a poor urinary output. The right atrial pressure was 8 mmHg and the left internal jugular catheter showed a pressure of 18 mmHg. Echocardiography was diagnostic of a mass blocking most of the right atrial cavity which was extending upward to the SVC. The patient was taken back for surgery which revealed localized cardiac tamponade compressing the right atrium, causing SVC syndrome. Evacuation of hematoma leads to rapid relief of symptoms [7].

SVC syndrome has been seen occurring in patients who had thoracic surgeries other than cardiac surgeries. A 53-year-old male who had esophagectomy for adenocarcinoma, reported by Hay et al., developed upper body swelling, hypotension, anuria, and metabolic acidosis within the first postoperative day. The venogram showed occlusion of the brachiocephalic vein and SVC by a thrombus. The symptoms resolved a week after intravenous anticoagulation was started [8].

The aorta, due to its structural proximity to SVC, can be a contributing factor to SVC obstruction. Aortic dissection and aortic aneurysm have been reported to cause SVC syndrome. Also, the ear is not the only structure where bleeding could be seen as a result of SVC syndrome. SVC obstruction can also cause bleeding in other areas. In a case reported by Bartek et al., a patient experienced intracranial hemorrhage secondary to intracranial hypertension caused by SVC syndrome. The SVC syndrome, in that case, was caused by an aortic aneurysm, treatment of which leads to the resolution of the intracranial hemorrhage [9].

Patient position may play an important role in ear bleeding during surgery. Trendelenburg position seems to increase the risk of having otorrhagia. This is due to increased pressure on the external ear vasculature due to gravity. Under that pressure, the subcutaneous capillaries rupture leading to external ear bleeding. Laparoscopic procedures can augment the effect of gravity by increasing the arterial and venous pressure even further. Two gynecologic laparoscopic surgery cases, published in a paper by Aunac and Nsengiyumva, reported otorrhagia after the surgeries and a case published by España Fuente et al. had bilateral external ear bleeding after laparoscopic left hemicolectomy [10,11]. All of these surgeries were performed in Trendelenburg position. Consistent with this theory, Larsen et al. suggest that reverse Trendelenburg position in neurosurgery decreases intracranial and jugular bulb pressure [12]. Chan et al. reported bilateral otorrhagia in a 60-year-old woman in Trendelenburg position [13].

Chan et al. mention that all five previously reported cases of intraoperative otorrhagia were in women and that female sex might be a risk factor for the condition [13]. Our patient, however, was male. It is very likely that the female preponderance is simply a coincidence since there is no compelling physiological mechanism to support it. Furthermore, epistaxis is more common in males [14]. The association of gender with otorrhagia cannot be determined based on the low number of reported cases, therefore, more cases need to be reported.

## Conclusions

Surgical factors leading to SVC obstruction include surgery type, technique, proximity to SVC, and patient positioning. Limited visibility of a patient’s face and body can delay the diagnosis. Echocardiography is instrumental in diagnosing the cause of SVC syndrome. Every effort should be made to keep the patient’s head adequately exposed to the anesthesia provider during surgery. Vigilance is required in monitoring the changes in the patient’s hemodynamics to detect SVC syndrome early. Prompt measures should be taken once alarming pressure changes and signs are detected to avoid morbidity.
